# A Novel Pyroptosis-related Prognostic Model for Hepatocellular Carcinoma

**DOI:** 10.3389/fcell.2021.770301

**Published:** 2021-11-15

**Authors:** Qianqian Wu, Sutian Jiang, Tong Cheng, Manyu Xu, Bing Lu

**Affiliations:** ^1^ Department of Clinical Biobank, Affiliated Hospital of Nantong University, Nantong, China; ^2^ Department of Medicine, Nantong University, Nantong, China

**Keywords:** Hepatocellular carcinoma, pyroptosis, prognostic model, risk score, tumor immunity

## Abstract

Hepatocellular carcinoma (HCC) is the second most lethal malignant tumor because of its significant heterogeneity and complicated molecular pathogenesis. Novel prognostic biomarkers are urgently needed because no effective and reliable prognostic biomarkers currently exist for HCC patients. Increasing evidence has revealed that pyroptosis plays a role in the occurrence and progression of malignant tumors. However, the relationship between pyroptosis-related genes (PRGs) and HCC patient prognosis remains unclear. In this study, 57 PRGs were obtained from previous studies and GeneCards. The gene expression profiles and clinical data of HCC patients were acquired from public data portals. Least absolute shrinkage and selection operator (LASSO) Cox regression analysis was performed to establish a risk model using TCGA data. Additionally, the risk model was further validated in an independent ICGC dataset. Our results showed that 39 PRGs were significantly differentially expressed between tumor and normal liver tissues in the TCGA cohort. Functional analysis confirmed that these PRGs were enriched in pyroptosis-related pathways. According to univariate Cox regression analysis, 14 differentially expressed PRGs were correlated with the prognosis of HCC patients in the TCGA cohort. A risk model integrating two PRGs was constructed to classify the patients into different risk groups. Poor overall survival was observed in the high-risk group of both TCGA (*p* < 0.001) and ICGC (*p* < 0.001) patients. Receiver operating characteristic curves demonstrated the accuracy of the model. Furthermore, the risk score was confirmed as an independent prognostic indicator via multivariate Cox regression analysis (TCGA cohort: HR = 3.346, *p* < 0.001; ICGC cohort: HR = 3.699, *p* < 0.001). Moreover, the single-sample gene set enrichment analysis revealed different immune statuses between high- and low-risk groups. In conclusion, our new pyroptosis-related risk model has potential application in predicting the prognosis of HCC patients.

## Introduction

Worldwide, primary liver cancer is the sixth most prevalent malignancy and the fourth leading cause of cancer-related death (Villanueva, 2019). Hepatocellular carcinoma (HCC), the most common type of primary liver cancer, usually develops after long-term chronic hepatitis, liver fibrosis, and cirrhosis (Sia et al., 2017). Despite significant advances in diagnosis and treatment over the past few decades, the prognosis of HCC remains unsatisfactory because of its extreme heterogeneity (Nault and Villanueva, 2015). The 5-years survival rate of HCC is only 18%, ranking second after pancreatic cancer in terms of lethality (Jemal et al., 2017) (Siegel et al., 2018). HCC is a complex and multistep disease involving genetic and epigenetic alterations. Currently, the prognostication of HCC is mainly based on clinicopathological staging systems, although multiple marker features, such as mutations in the well-known *TP53* gene and the expression of cellular proliferation-related genes, have been identified to predict survival (Li et al., 2018) (Schulze et al., 2015) (Totoki et al., 2014). Therefore, novel prognostic biomarkers are urgently needed to predict survival and outline individualized treatment plans for HCC patients.

Pyroptosis, a form of gasdermin-mediated programmed cell death activated by inflammasomes, occurs in vertebrates as an innate immune response mechanism (Wallach et al., 2016; Shi et al., 2017). Reduced pathogen clearance efficiency and adaptive immune response dysfunction may be caused by the dysregulation of pyroptosis, resulting in tissue damage. Increasing evidence has revealed that pyroptosis plays a role in the occurrence and progression of diverse human diseases, including malignant tumors. More recently, several studies demonstrated that the pyroptosis of tumor cells could be induced chemically *in vitro* and *in vivo* (Rébé et al., 2015). Because activating pyroptosis stimulates the release of multiple inflammatory mediators that may promote cancer progression, such as IL-1 and IL-18, some researchers consider pyroptosis a protumorigenic mechanism (Dunn et al., 2012) (Hu et al., 2010) (Tang et al., 2020). Nevertheless, recent research has confirmed that exogenously activated pyroptosis induces strong antitumor activity (Wang et al., 2019) (Xia et al., 2019).

Here, we explored the mRNA expression level of pyroptosis-related genes (PRGs) and their relationship with the survival of HCC patients to identify differentially expressed prognostic genes using TCGA data. We constructed a risk-coefficient model consisting of two genes: *GSDME* and *PLK1.* The prognostic model was further validated using an ICGC cohort. Importantly, the model shows a promising predictive ability and may be used in clinical decision making for HCC patients.

## Materials and Methods

### Data Acquisition

The gene expression profile and clinical data of 371 HCC cases were acquired from the TCGA database up to September 3, 2020 and processed into a matrix file with Perl programming language. The gene expression data and corresponding clinicopathological data of another 231 HCC cases were acquired from the ICGC database (https://dcc.icgc.org/projects/LIRI-JP) as a validation cohort. Additionally, 57 genes related to pyroptosis from a previous study and GeneCards (https://www.genecards.org/) were collected and shown in Supplementary Table S1 (Xia et al., 2019).

### Identification of Differentially Expressed PRGs

Differentially expressed PRGs between tumor and non-tumor tissues in the TCGA cohort were screened with a threshold false discovery rate (FDR) < 0.05 using the “limma” R package. Cases without expression data for most genes were excluded.

### Signature Construction

The “survival” R package was used to perform univariate Cox analysis in TCGA cases to study the prognostic value of PRGs. Then, differentially expressed prognostic PRGs were selected as candidate genes for further analysis. The correlation networks to statuses between these candidate genes were analyzed via the “igraph” R package. Least absolute shrinkage and selection operator (LASSO) cox regression analysis was conducted in the TCGA cohorts to build a prognostic model of PRGs using 10-fold cross validation with the “glmnet” R package. Subsequently, individualized risk scores were obtained based on the mRNA expression of selected genes and their regression coefficients estimated in the LASSO Cox regression analysis. The risk score of each HCC patient was calculated with the following formula:

$$Risk\;score=\sum_{i=1}^{n}\left(Exp_{i}*Coe_{i}\right)$$

The median risk score was utilized to classify patients into high- and low-risk groups.

### Evaluation of the Prognostic Signature

The “stats” and “Rtsne” R packages were used to conduct principal component analysis (PCA) and t-distributed stochastic neighbor embedding (t-SNE), respectively, to study the distribution of different risk groups. Univariate Cox regression and multivariate Cox regression analyses were conducted to evaluate the independent prognostic value of the signature using the “survival” R package. The variables included in the univariate Cox regression analysis were age, gender, grade, TNM stage, and risk score. Variables with *p* < 0.05 in the univariate Cox regression were included in the multivariate Cox regression analysis. The Kaplan–Meier method and log-rank test were performed to compare overall survival (OS) between high-risk and low-risk groups. Additionally, receiver operator characteristic (ROC) curves plotted with the “timeROC” R package were applied to assess the accuracy of the risk model.

### Functional Enrichment Analysis

Gene ontology (GO) and Kyoto Encyclopedia of Genes and Genomes (KEGG) pathway analyses were performed using the “clusterProfiler” R package. Moreover, single-sample gene set enrichment analysis (ssGSEA) was applied to score the infiltrating levels of diverse immune cells and the activity levels of multiple immune-related pathways via the “gsva” R package. *p* values < 0.05 and *q* values < 0.05 were used as the threshold.

### Statistical Analysis

All statistical analyses were performed using R software (Version 4.0.2). A *p* value < 0.05 was considered statistically significant.

## Results

### Differentially Expressed PRGs in the TCGA Cohort and Their Functional Analysis

**FIGURE 1:**
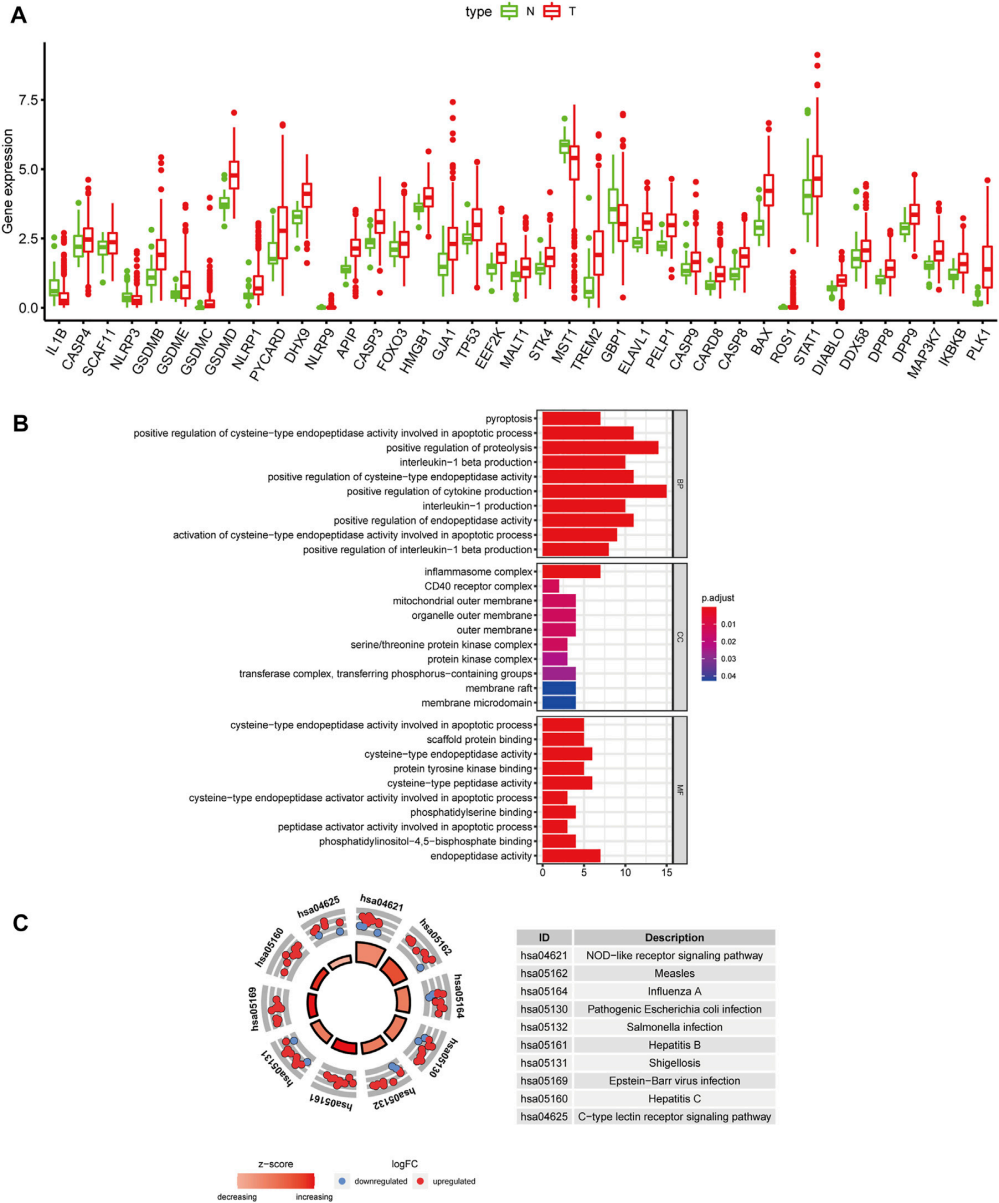
Differentially expressed PRGs and functional enrichment analysis. **(A)** Expression status of 39 differentially expressed PRGs in HCC and adjacent normal samples. Red and green represent tumor (T) and adjacent normal tissues (N), respectively. **(B)** GO functional analysis of 39 differentially expressed PRGs (BP: biological processes, CC: cellular components, MF: molecular function). **(C)** KEGG pathway analysis of 39 differentially expressed PRGs.

We obtained 57 PRGs from a previous study and analyzed the expression level of these genes in 371 HCC patients from TCGA (Xia et al., 2019). Based on the screening criteria of FDR<0.05, 39 PRGs were significantly differentially expressed between tumor and normal liver tissues. As shown in the boxplot, *IL1B*, *MST1, GBP1,* and *NLRP3* were downregulated, and the other 35 PRGs were upregulated in tumor tissues (Figure 1A). Additionally, GO and KEGG analyses were performed to study the biological functions of these differentially expressed PRGs. The results of GO functional analysis revealed that the positive regulation of cytokine production, inflammasome complex, and endopeptidase activity involved in apoptotic processes were the most enriched GO terms in biological process, cellular component, and molecular function categories, respectively (Figure 1B). In the KEGG pathway analysis, the PRGs were mainly enriched in pyroptosis-related pathways, such as the NOD-like receptor signaling pathway (Fritz et al., 2006). Moreover, the PRGs were identified to participate in the C-type lectin receptor signaling pathway and pathways related to bacterial and viral infections, including hepatitis B and C infections (Figure 1C).

### Construction of the Predictive Signature in the TCGA Cohort

**FIGURE 2:**
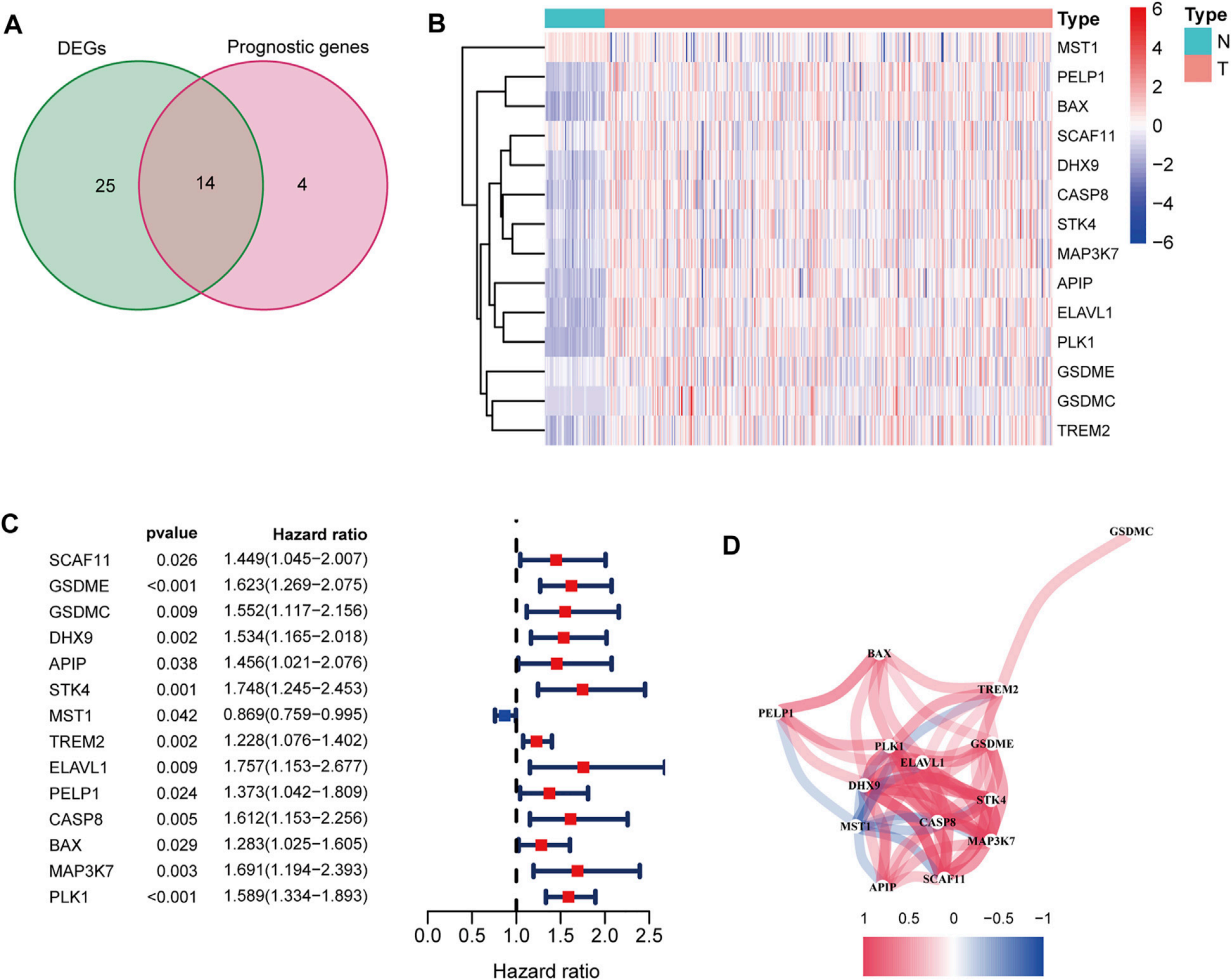
Identification of candidate PRGs in the TCGA cohort. **(A)** Venn diagram to screen 14 differentially expressed PRGs related to the OS of HCC patients (DEGs: differentially expressed genes). **(B)** Heatmap showed that *MSTG1* was downregulated, and the other 13 intersection genes were upregulated in HCC. **(C)** Forest plots showed that the intersection genes were all significantly correlated with the OS of HCC patients according to the univariate Cox regression analysis. **(D)** Correlation network between the intersection genes. Different colors represent different correlation coefficients.

**FIGURE 3:**
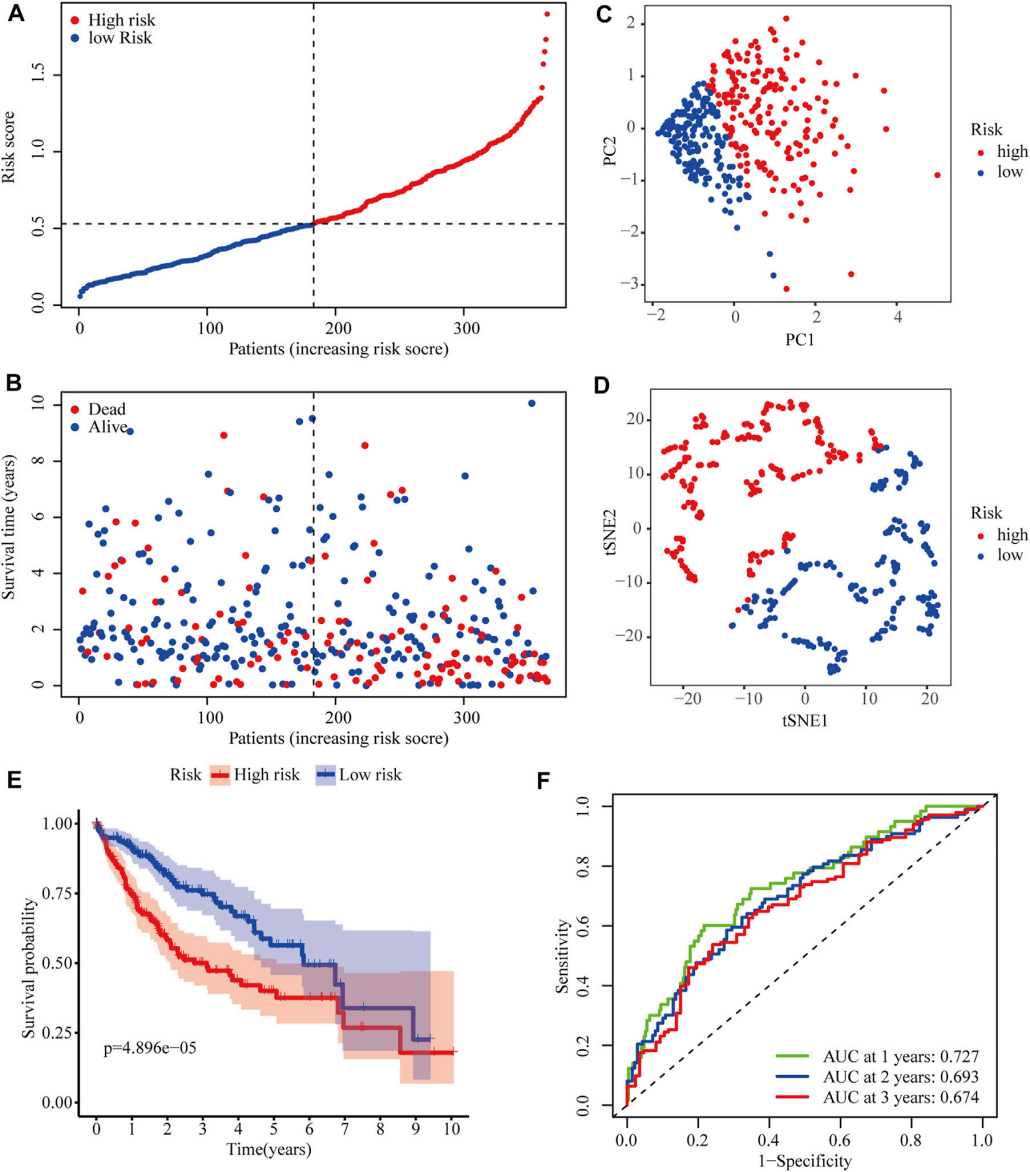
Predictive performance of the two-PRG model in the TCGA cohort. **(A)** Risk score curve shows the distribution of the model and the median score. **(B)** Distribution of survival statuses and risk scores in the TCGA cohort. **(C)** Principal component analysis (PCA) results in the TCGA cohort. **(D)** t-distributed stochastic neighbor embedding (tSNE) results in the TCGA cohort. **(E)** Kaplan–Meier curves show the OS of patients in different risk groups. **(F)** ROC curves were used to evaluate the predictive power of the model in the TCGA cohort (AUC: area under the curve).

After excluding cases without survival information and those with a follow-up time of 0 days, univariate Cox regression analyses were carried out to assess the correlations between PRG expression and OS in 365 HCC patients in the TCGA cohort. The results showed that 18 PRGs were significantly correlated with the prognosis of HCC patients (*p* < 0.05) (Supplementary Figure S1). The clinicopathological characteristics of the patients involved in our study were analyzed in Table 1. We selected 14 PRGs that were differentially expressed and significantly correlated with the OS of HCC patients (Figures 2A–C). A correlation network between these genes was established and presented in Figure 2D. Then, LASSO Cox regression analysis was conducted to identify the markers with the best predictive performance and calculate the regression coefficient. As a result, *GSDME* and *PLK1* were identified to establish a risk model. The risk score for each patient was calculated as follows: Risk score = 0.1475 × expression level of *GSDME* + 0.2859 × expression level of *PLK1*. Based on the median risk score, HCC patients were divided into high- and low-risk groups (Figure 3A). With increasing risk scores, the incidence of death increased (Figure 3B). PCA and t-SNE results demonstrated that patients with different risk statuses were clustered in two areas (Figures 3C,D). Additionally, Kaplan–Meier survival curves revealed that patients with high risk scores exhibited a poorer OS than those with low risk scores (*p* < 0.001) (Figure 3E). Time-dependent ROC curves were generated to evaluate the accuracy of the model in predicting the prognosis of HCC patients in the TCGA cohort, and the areas under the curves (AUCs) were 0.727, 0.693, and 0.674 at 1 year, 2 years, and 3 years, respectively (Figure 3F).

**TABLE 1:** Clinicopathological characteristics of HCC patients involved in the study.

Characteristic	TCGA cohort	ICGC cohort
Total	365	231
Age		
Median	61	69
Rage	16–90	31–89
Gender		
Female	34(32.60%)	61 (26.41%)
Male	43(67.40%)	170 (73.59%)
Grade		
Grade 1	55 (15.07%)	NA
Grade 2	175 (47.95%)	NA
Grade 3	118 (32.33%)	NA
Grade 4	12 (3.28%)	NA
Unknown	5 (1.37%)	NA
Stage		
Ⅰ	170 (46.57%)	36(15.58%)
Ⅱ	84 (23.01%)	105(45.45%)
Ⅲ	83 (22.74%)	71(30.74%)
Ⅳ	4 (1.10%)	19(8.23%)
Unknown	24 (6.58%)	0(0.00%)
T		
T1	180 (49.32%)	NA
T2	91 (24.93%)	NA
T3	78 (21.37%)	NA
T4	13 (3.56%)	NA
Unknown	3(0.82%)	NA
N		
N0	248(67.94%)	NA
N1	4(1.10%)	NA
Unknown	113(30.96%)	NA
M		
M0	263(72.06%)	NA
M1	3 (0.82%)	NA
Unknown	99(27.12%)	NA

### Further Validation of the Model in the ICGC Cohort

**FIGURE 4:**
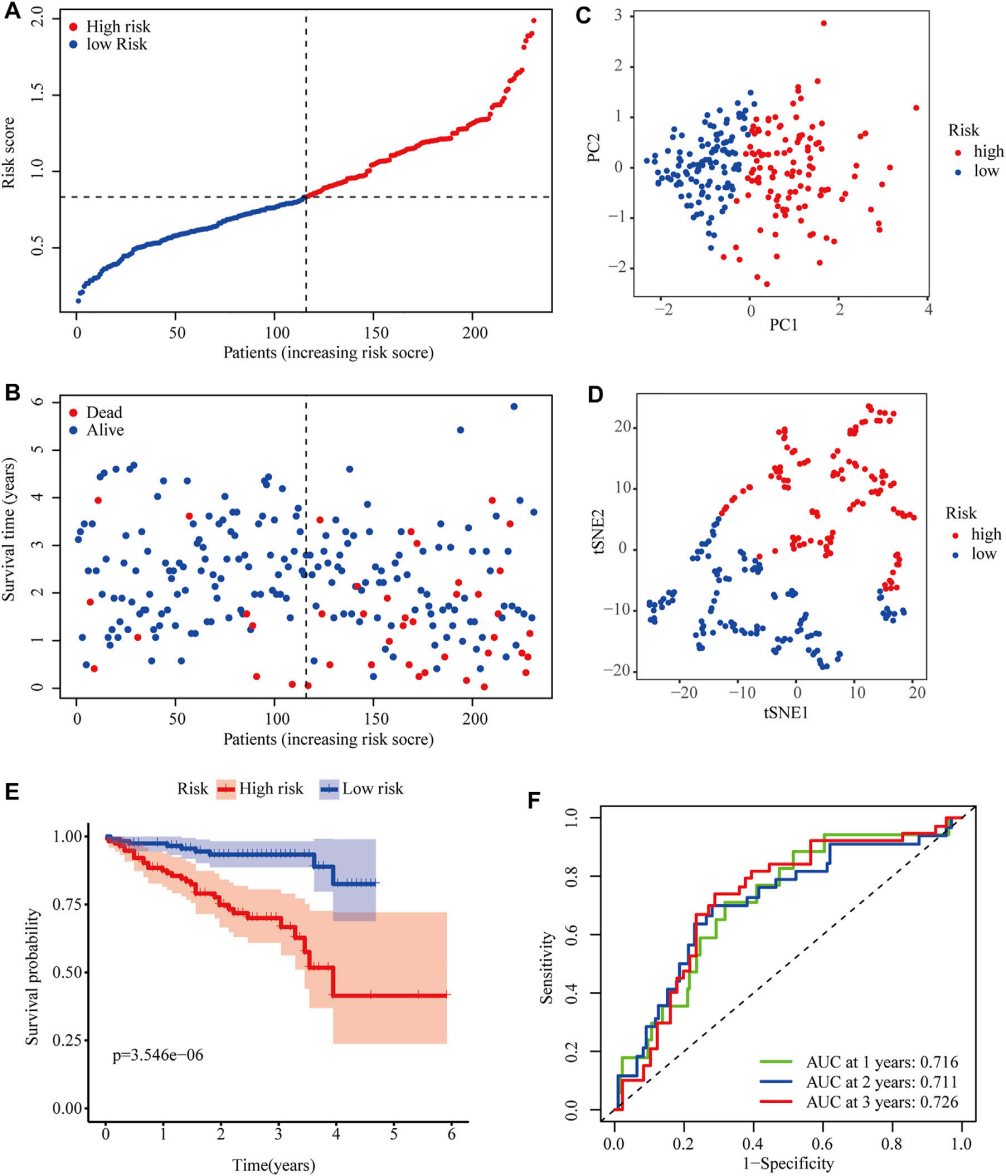
Predictive performance of the two-PRG model in the ICGC cohort. **(A)** Risk score curve shows the distribution of the model and the median score. **(B)** Distribution of survival statuses and risk scores. **(C)** Principal component analysis (PCA) results in the ICGC cohort. **(D)** t-distributed stochastic neighbor embedding (tSNE) results in the ICGC cohort. **(E)** Kaplan–Meier curves show the OS of patients in different risk groups. **(F)** ROC curves were used to evaluate the predictive power of the model in the ICGC cohort (AUC: area under the curve).

To further evaluate the robustness of the two-gene signature established in the TCGA cohort, the risk scores of HCC patients in the ICGC cohort were also calculated according to the same formula. Then, ICGC cases were classified into high- and low-risk groups according to the median risk score (Figure 4A). Similarly, the number of deaths increased with higher risk scores (Figure 4B). The classifying ability of the risk score was confirmed by PCA and t-SNE analysis (Figures 4C,D). Consistently, Kaplan–Meier plots showed that high risk scores were also significantly correlated with poor OS in the ICGC cohort (*p* < 0.001) (Figure 4E). The AUCs of 0.716 at 1 year, 0.711 at 2 years, and 0.726 at 3 years obtained from ROC curve analyses demonstrated that the model accurately predicted the prognosis of HCC patients from the ICGC database (Figure 4F).

### The Independent Predictive Ability of the Model

**FIGURE 5:**
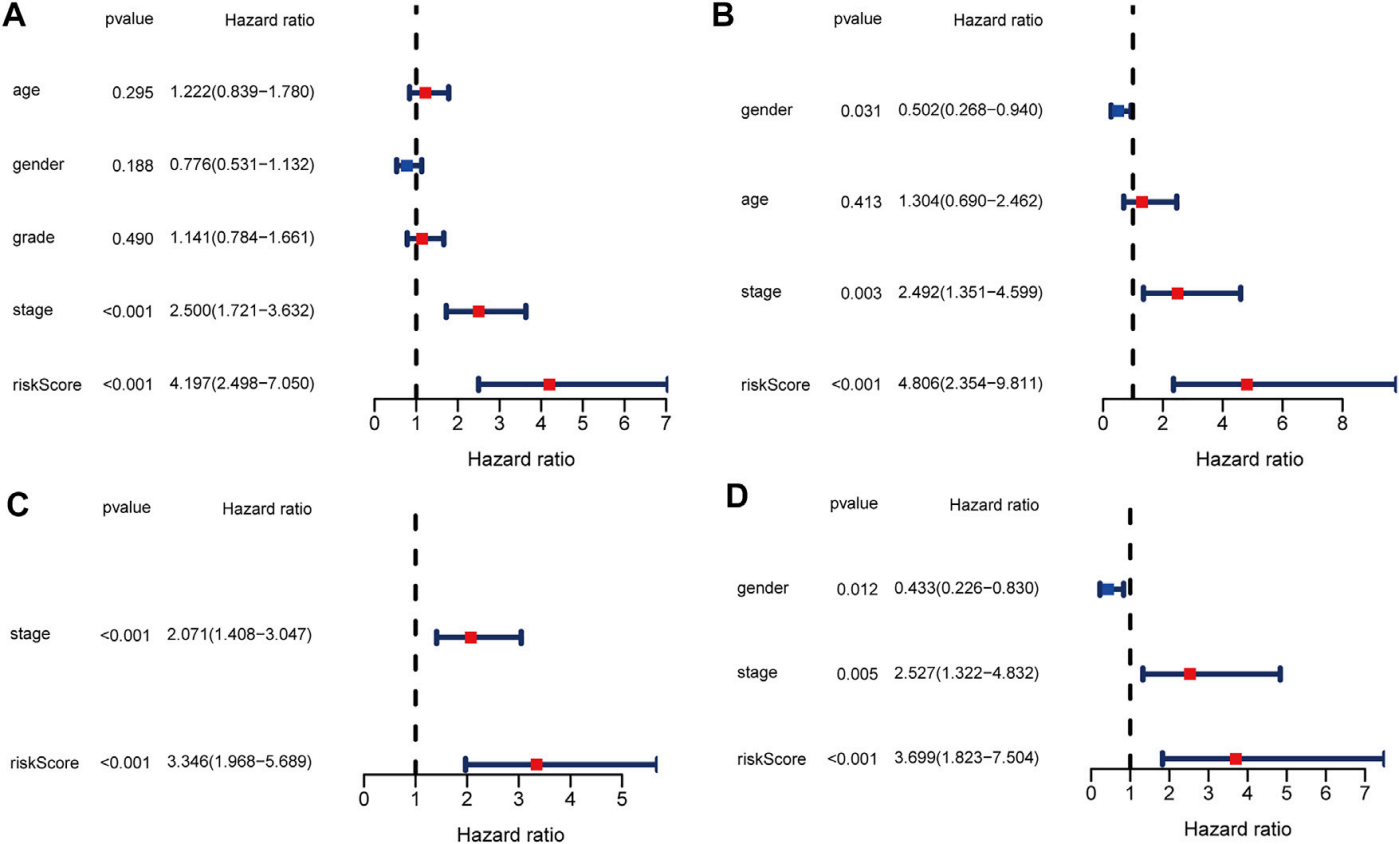
Univariate and multivariate Cox regression analysis in TCGA and ICGC cohorts. **(A)** Forest plot of the univariate regression analysis in the TCGA cohort. **(B)** Forest plot of the univariate regression analysis in the ICGC cohort. **(C)** Forest plot of the multivariate regression analysis in the TCGA cohort. **(D)** Forest plot of the multivariate regression analysis in the ICGC cohort.

To evaluate the ability of the signature as an independent prognostic indicator, multiple variables were included in univariate and multivariate Cox regression analyses. As a result, univariate Cox regression analysis revealed that a higher risk score predicted the OS of HCC patients in both TCGA and ICGC cohorts (HR = 4.197, 95% CI:2.498–7.050, *p* < 0.001; HR = 4.806, 95% CI: 4.354–9.811, *p* < 0.001, respectively) (Figures 5A,B). In the multivariate analysis, the risk score was further demonstrated to be an independent predictor for TCGA and ICGC patients (HR = 3.346, 95% CI: 1.968–5.689, *p* < 0.001; HR = 3.699, 95% CI: 1.823–7.504, *p* < 0.001, respectively) (Figures 5C,D).

### Functional Analysis Based on the Model in TCGA and ICGC Cohorts

**FIGURE 6:**
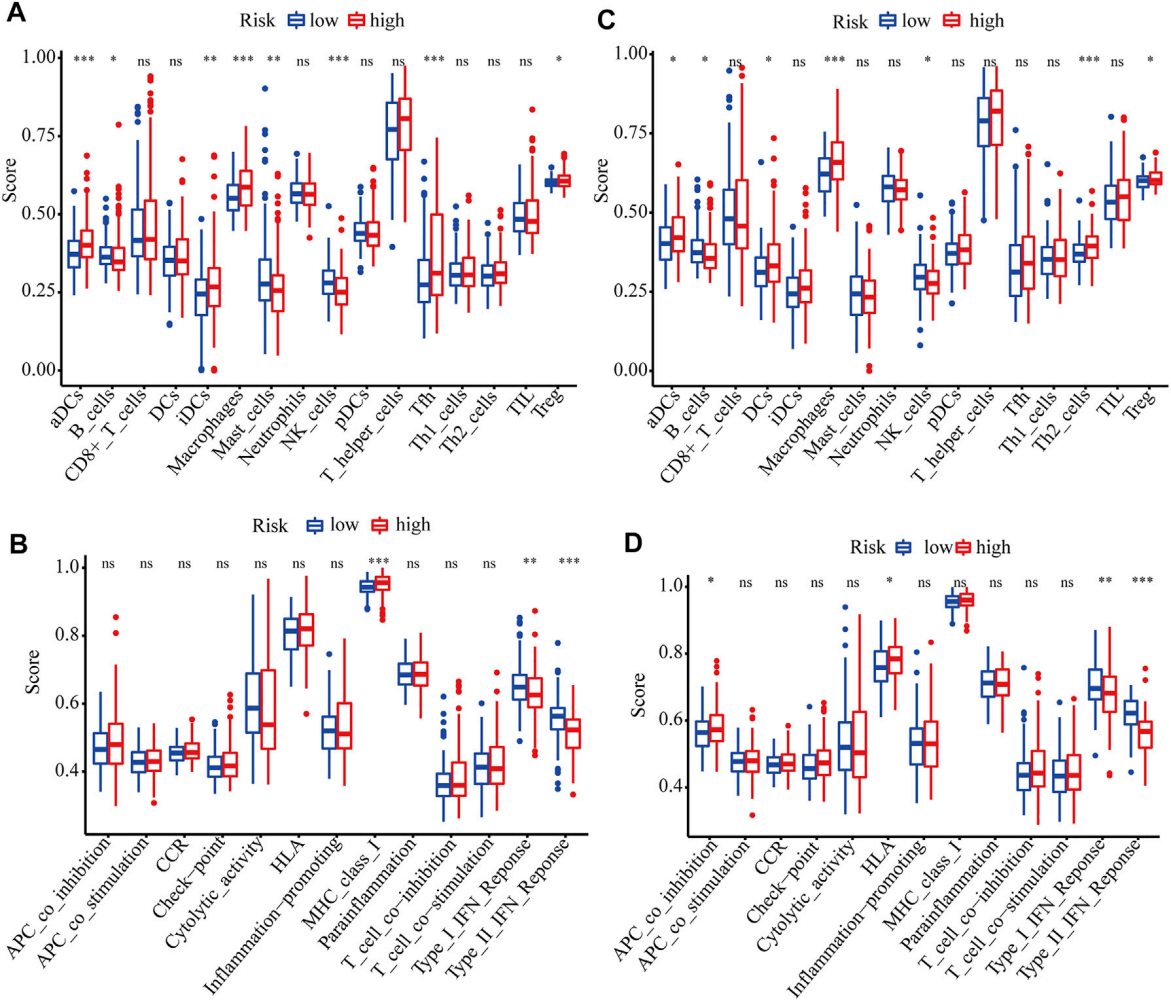
ssGSEA scores of different risk groups in TCGA and ICGC cohorts. **(A, B)** Boxplots show the scores of immune cells and immune-related functions in different risk groups in the TCGA cohort. **(C, D)** Boxplots show the scores of immune cells and immune-related functions in different risk groups in the ICGC cohort. *, P < 0.05; **, P < 0.01; ***, P < 0.001; ns, not significant.

GO and KEGG analyses were performed to analyze the biological functions related to the risk model. The differentially expressed genes between high- and low-risk groups were included in the analysis. The top 5 GO terms and KEGG results were presented in Table 2. These genes were enriched in several functions related to the cell cycle, such as mitotic nuclear division, nuclear division, and chromosome segregation. Tumor immunity is an important factor affecting cancer progression. Thus, we also investigated the correlation between the risk model and tumor immunity. The levels of immune cells and immune-related pathways were quantified with ssGSEA. In the TCGA cohort, the scores for aDCs, iDCs, macrophages, Tfh cells, Tregs, and MHC class I were significantly higher, whereas B cells, mast cells, NK cells, type I IFN response, and type II IFN response were significantly lower in the high-risk group than in the low-risk group (Figures 6A,B). In the ICGC cohort, the scores for aDCs, DCs, macrophages, Th2 cells, Tregs, APC co-inhibition, and HLA were significantly higher, whereas B cells, NK cells, type I IFN response, and type II IFN response were significantly lower in the high-risk group than in the low-risk group (Figures 6C,D).

**TABLE 2:** GO and KEGG analysis results in TCGA and ICGC cohorts based on differentially expressed PRGs.

Category	TCGA			ICGC		
	ID	Terms	*p*-value	ID	Description	*p*-value
BP	GO:0140014	Mitotic nuclear division	3.42E-30	GO:0140014	Mitotic nuclear division	5.93E-20
BP	GO:0000280	Nuclear division	3.43E-27	GO:0000280	Nuclear division	3.29E-17
BP	GO:0007059	Chromosome segregation	1.07E-25	GO:0007059	Chromosome segregation	2.90E-16
BP	GO:0048285	Organelle fission	1.14E-25	GO:0000070	Mitotic sister Chromatid segregation	3.68E-16
BP	GO:0000070	Mitotic sister Chromatid segregation	2.26E-24	GO:0048285	Organelle fission	2.11E-15
**CC**	GO:0098687	Chromosomal region	1.28E-23	GO:0062023	Collagen-containing extracellular matrix	3.91E-15
**CC**	GO:0000793	Condensed chromosome	4.64E-23	GO:0000779	Condensed chromosome, centromeric region	7.63E-14
**CC**	GO:0000775	Chromosome, centromeric region	8.32E-22	GO:0000775	Chromosome, centromeric region	2.10E-13
**CC**	GO:0000779	Condensed chromosome, centromeric region	1.54E-21	GO:0000793	Condensed chromosome	4.30E-13
**CC**	GO:0005819	Spindle	4.25E-20	GO:0098687	Chromosomal region	1.89E-12
MF	GO:0003688	DNA replication origin binding	2.86E-10	GO:0008395	Steroid hydroxylase activity	2.39E-10
MF	GO:0140097	Catalytic activity, acting on DNA	4.34E-09	GO:0008514	Organic anion Transmembrane transporter activity	1.80E-09
MF	GO:0017116	Single-stranded DNA helicase activity	9.14E-09	GO:0020037	Heme binding	2.67E-09
MF	GO:0003678	DNA helicase activity	5.67E-08	GO:0046906	Tetrapyrrole binding	3.66E-09
MF	GO:0008094	DNA-dependent ATPase activity	1.23E-07	GO:0004497	Monooxygenase activity	4.98E-09
KEGG pathway	hsa04110	Cell cycle	5.58E-23	hsa04110	Cell cycle	6.60E-14
KEGG pathway	hsa03030	DNA replication	1.81E-14	hsa00980	Metabolism of xenobiotics by cytochrome P450	9.00E-13
KEGG pathway	hsa03430	Mismatch repair	1.16E-05	hsa00982	Drug metabolism - cytochrome P450	5.90E-12
KEGG pathway	hsa00010	Glycolysis/Gluconeogenesis	3.02E-05	hsa00830	Retinol metabolism	5.37E-10
KEGG pathway	hsa04114	Oocyte meiosis	3.08E-05	hsa05204	Chemical carcinogenesis	1.18E-09

## Discussion

HCC is the second most lethal malignant tumor because of its extreme heterogeneity and complicated molecular pathogenesis (Nault and Villanueva, 2015). Accurately predicting the OS of HCC patients is of great importance for clinical decision making. However, there are currently no effective and reliable prognostic biomarkers for HCC patients. Thus, establishing a robust prediction model and identifying effective biomarkers to predict the outcomes of HCC patients are urgently needed.

We collected 57 genes related to pyroptosis from a previous study. Based on a systematical analysis using a TCGA dataset, 39 differentially expressed PRGs between HCC and non-tumor tissues were identified. According to GO functional and KEGG pathways analyses, these genes are primarily related to pyroptosis. Then, univariate and LASSO Cox regression analyses were carried out to establish an mRNA-based risk model to predict the prognosis of HCC. The model includes two PRGs: *GSDME* and *PLK1*. According to the median risk score, patients with HCC were classified into different risk groups, and patients in the high-risk group exhibited a significantly poorer prognosis. The results of ROC curve analyses indicated the predictive performance of this prognostic model. Moreover, the risk model was demonstrated to be independent of other clinicopathological factors in HCC. More importantly, we further validated the model using an independent ICGC dataset. To our satisfaction, the model was confirmed to be an independent risk factor and exhibited an excellent predictive power in the ICGC cohort.

*GSDME*, a gene related to hereditary hearing loss, has been reported to be involved in various cancers in the past few decades (Laer et al., 1998) (De Schutter et al., 2020) (de Beeck et al., 2011). Recently, increasing evidence confirmed that GSDME functions as a pore-forming effector molecule and is activated after caspase-3-mediated cleavage, leading to secondary necrosis after apoptosis or primary necrosis termed pyroptosis without apoptosis (Broz et al., 2020) (Rogers et al., 2017) (Wang et al., 2018) (Wang et al., 2017). Differential methylation patterns of *GSDME* have been identified between tumor and normal tissues in specific cancer types. Ibrahim et al. reported that *GSDME* methylation exhibited potential as a prognostic biomarker of colorectal cancer and pan-cancer (Ibrahim et al., 2019a) (Ibrahim et al., 2019b). Croes et al. showed that *GSDME* methylation was strongly correlated with the prognosis of breast cancer patients (Croes et al., 2018). In the current study, we revealed that *GSDME* mRNA expression could predict the survival of HCC patients. As a pivotal molecule of mitosis and cytokinesis, PLK1 maintains genome stability in eukaryotic cells (Combes et al., 2017) (Liu et al., 2010). Cell cycle dysregulation is known to contribute to cancer. Therefore, it is not surprising that PLK1 expression is increased in various human malignant tumors, such as breast cancer, colorectal cancer, and melanoma, and PLK1 upregulation is associated with the poor prognosis of cancer patients (Takai et al., 2005) (Gutteridge et al., 2016). Wu et al. reported that combined treatment with the PLK1 inhibitor BI2536 and DDP at low doses induced pyroptosis via the caspase-3/GSDME axis in esophageal squamous cell carcinoma (Wu et al., 2019). Here, we revealed that the mRNA expression of *PLK1* is also increased in HCC and significantly associated with the poor prognosis of HCC patients.

Although several researchers have studied the relationship between pyroptosis and human cancer, there are few reports on its correlation with tumor immunity. An important aspect of our study is that we explored the correlations between the risk model and tumor immunity in HCC patients. Interestingly, we found that the infiltration levels of macrophages and Tregs were significantly increased in the high-risk group in both the training and validation cohorts. Previous studies have confirmed that increased infiltration levels of Tregs and tumor-associated macrophages, which promote immune invasion, are correlated with the poor prognosis of HCC patients (Liang et al., 2020) (Fu et al., 2007) (Zhou et al., 2016). Furthermore, NK cells, type I IFN response, and type II IFN response were significantly lower in the high-risk group. Studies have shown that the number of NK cells in tumor tissues is positively related to the survival of HCC patients (Sun et al., 2015). IFNs have been identified as pivotal factors in coordinating the interactions between tumors and the immune system (Dunn et al., 2006). Therefore, we speculate that impaired antitumor immunity may contribute to the poorer prognosis of patients with high-risk scores.

To date, this is the first study to investigate the relationships between a considerable number of PRGs and the prognosis of HCC patients. We obtained multiple prognostic PRGs and constructed a novel pyroptosis-related prognostic model. However, there are still some limitations to our study. First, the prognostic signature was established and validated using retrospective data, and its clinical applicability needs to be verified with prospective data. Second, the underlying biological functions and specific molecular mechanisms of the two genes in combination require further examination. Third, the correlations between the risk score and tumor immunity were not experimentally proven.

In summary, we constructed and validated a novel risk model consisting of two PRGs. The model demonstrated independent prognostic value, thereby providing important insight into the survival prediction of HCC. However, further verification and mechanistic exploration are indispensable in the future.

## Data Availability

The datasets presented in this study can be found in online repositories. The names of the repository/repositories and accession number(s) can be found in the article/Supplementary Material.

## Supplementary Material

Supplementary Figure S1 PRGs significantly correlated with the OS of HCC patients in the univariate Cox regression analysis.

## Abbreviations

aDC, activated dendritic cell; APC, antigen presenting cell; AUC, area under the curve; CI, confidence interval; FDR, false discovery rate; GO, gene ontology; HCC, hepatocellular carcinoma; HR, hazard ratio; HLA, human leukocyte antigen; ICGC, International Cancer Genome Consortium; iDC, immature dendritic cell; KEGG, Kyoto Encyclopedia of Genes and Genomes; LASSO, least absolute shrinkage and selection operator; MHC, major histocompatibility complex; OS, overall survival; PCA, principal component analysis; PRGs, pyroptosis-related genes; ROC, receiver operating characteristic; ssGSEA, single-sample gene set enrichment analysis; TCGA, The Cancer Genome Atlas; t-SNE, t-distributed stochastic neighbor embedding; Tfh, T follicular helper cell; Treg, regulatory T cells; Th2, T helper 2 cell.
