# Supplementary material for: A Novel Pyroptosis-related Prognostic Model for Hepatocellular Carcinoma
Source: Front Cell Dev Biol. 2021 Nov 15;9:770301. doi: 10.3389/fcell.2021.770301 (PMC8634647; doi:10.3389/fcell.2021.770301)
Supplement: Supplementary file 1 [file Table1.DOCX]

**Supplementary table 1**: 57 pyroptosis-related genes included in this study.

| **GENE** | **Description** |
| --- | --- |
| AIM2 | Absent In Melanoma 2 |
| APIP | APAF1 Interacting Protein |
| BAX | BCL2 Associated X, Apoptosis Regulator |
| CARD8 | Caspase Recruitment Domain Family Member 8 |
| CASP1 | Caspase 1 |
| CASP3 | Caspase 3 |
| CASP4 | Caspase 4 |
| CASP5 | Caspase 5 |
| CASP8 | Caspase 8 |
| CASP9 | Caspase 9 |
| CPTP | Ceramide-1-Phosphate Transfer Protein |
| DDX3X | DEAD-Box Helicase 3 X-Linked |
| DDX58 | DExD/H-Box Helicase 58 |
| DHX9 | DExH-Box Helicase 9 |
| DIABLO | Diablo IAP-Binding Mitochondrial Protein |
| DPP8 | Dipeptidyl Peptidase 8 |
| DPP9 | Dipeptidyl Peptidase 9 |
| EEF2K | Eukaryotic Elongation Factor 2 Kinase |
| ELAVL1 | ELAV Like RNA Binding Protein 1 |
| FGF21 | Fibroblast Growth Factor 21 |
| FOXO3 | Forkhead Box O3 |
| GBP1 | Guanylate Binding Protein 1 |
| GJA1 | Gap Junction Protein Alpha 1 |
| GSDMA | Gasdermin A |
| GSDMB | Gasdermin B |
| GSDMC | Gasdermin C |
| GSDMD | Gasdermin D |
| GSDME | Gasdermin E |
| GZMA | Granzyme A |
| HMGB1 | High Mobility Group Box 1 |
| IKBKB | Inhibitor Of Nuclear Factor Kappa B Kinase Subunit Beta |
| IL18 | Interleukin 18 |
| IL1B | Interleukin 1 Beta |
| MALT1 | MALT1 Paracaspase |
| MAP3K7 | Mitogen-Activated Protein Kinase Kinase Kinase 7 |
| MST1 | Macrophage Stimulating 1 |
| NAIP | NLR Family Apoptosis Inhibitory Protein |
| NFKB1 | Nuclear Factor Kappa B Subunit 1 |
| NLRC4 | NLR Family CARD Domain Containing 4 |
| NLRP1 | NLR Family Pyrin Domain Containing 1 |
| NLRP3 | NLR Family Pyrin Domain Containing 3 |
| NLRP9 | NLR Family Pyrin Domain Containing 9 |
| P2RX7 | Purinergic Receptor P2X 7 |
| PELP1 | Proline, Glutamate And Leucine Rich Protein 1 |
| PLK1 | Polo Like Kinase 1 |
| PYCARD | PYD And CARD Domain Containing |
| PLAAT4 | Phospholipase A And Acyltransferase 4 |
| ROS1 | ROS Proto-Oncogene 1, Receptor Tyrosine Kinase |
| MAPK8 | Mitogen-Activated Protein Kinase 8 |
| SCAF11 | SR-Related CTD Associated Factor 11 |
| SCGB3A2 | Secretoglobin Family 3A Member 2 |
| STAT1 | Signal Transducer And Activator Of Transcription 1 |
| STK4 | Serine/Threonine Kinase 4 |
| TET2 | Tet Methylcytosine Dioxygenase 2 |
| TNF | Tumor Necrosis Factor |
| TP53 | Tumor Protein P53 |
| TREM2 | Triggering Receptor Expressed On Myeloid Cells 2 |
