# Supplementary figures and images for: A Novel Pyroptosis-related Prognostic Model for Hepatocellular Carcinoma
Source: Front Cell Dev Biol. 2021 Nov 15;9:770301. doi: 10.3389/fcell.2021.770301 (PMC8634647; doi:10.3389/fcell.2021.770301)

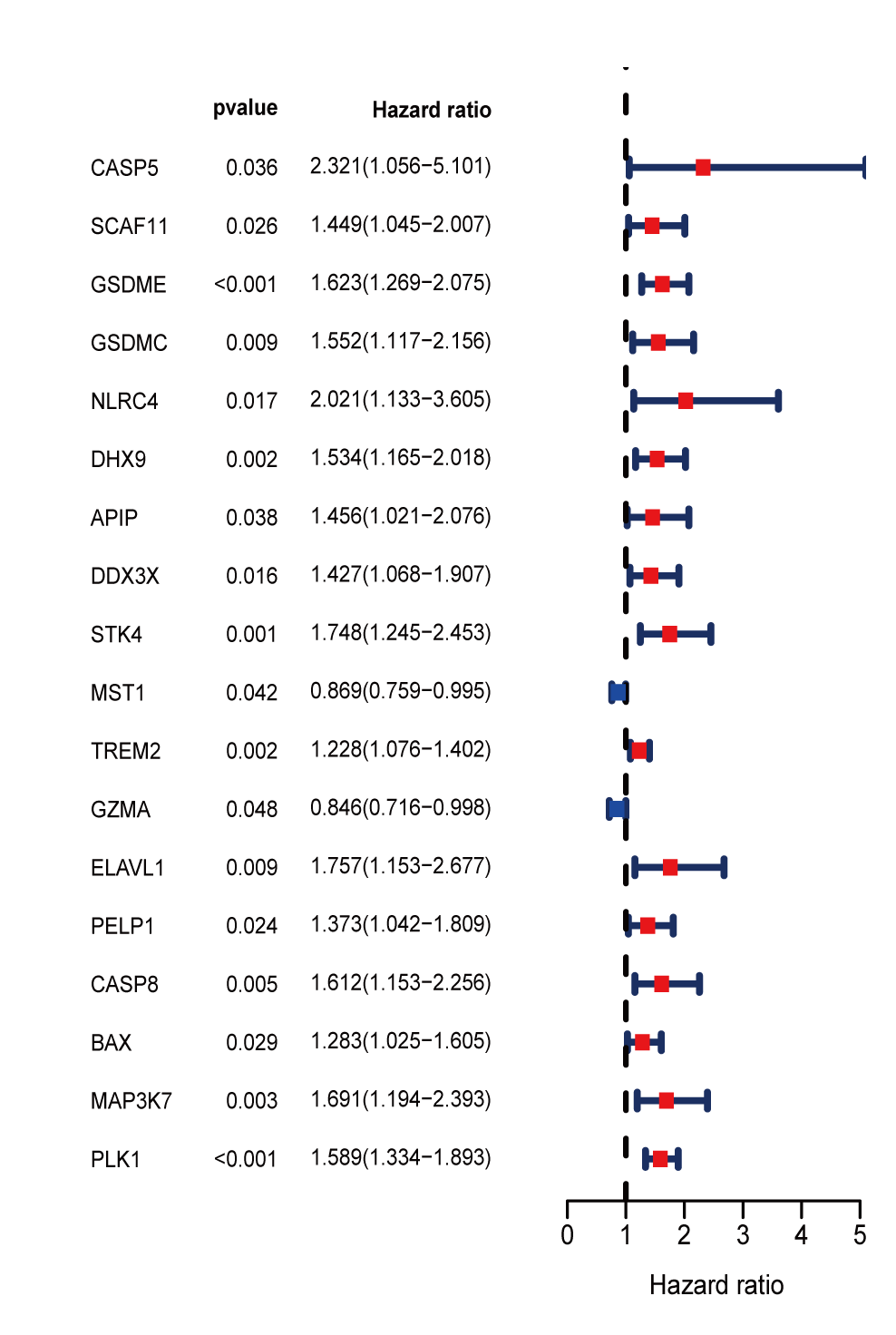

Supplement: Supplementary file 2 [file Image1.TIF]
